# Intake of eggshell membrane enhances bone mass and suppresses bone marrow adiposity in normal growing rats

**DOI:** 10.1016/j.bonr.2025.101840

**Published:** 2025-04-01

**Authors:** Nao Yashima, Kaoru Fujikawa, Wataru Minamizono, Hiroya Matsunaga, Jiazheng Lyu, Hirai Suito, Takumi Okunuki, Shingo Nakai, Masafumi Ohsako

**Affiliations:** a Graduate School of Health and Sports Science, Toyo University, 1-7-11 Akabanedai, Kita-ku, Tokyo 115-8650, Japan; b Department of Oral Anatomy, Showa Medical University School of Dentistry, 1-5-8 Hatanodai, Shinagawa-ku, Tokyo 142-0064, Japan; c Graduate School of Human Life Design, Toyo University, 1-7-11 Akabanedai, Kita-ku, Tokyo 115-8650, Japan; d Department of Anatomy, Teikyo University School of Medicine, 2-11-1 Kaga, Itabashi-ku, Tokyo 173-8605, Japan; e Research Organization of Science and Technology, Ritsumeikan University/Japan Society for the Promotion of Science, 5-3-1 Kojimachi, Chiyoda-ku, Tokyo 102-0083, Japan; f Department of Judo Seifuku and Health Sciences, Tokoha University Faculty of Health Promotional Sciences, 1230 Miyakoda-cho, Hamana-ku, Hamamatsu-shi, Shizuoka 431-2102, Japan; g Department of Health and Sports Science, Toyo University School of Health and Sports Science, 1-7-11 Akabanedai, Kita-ku, Tokyo 115-8650, Japan

**Keywords:** Eggshell membrane, Bone marrow adiposity, Bone mass, Osteoblast, Adipocyte, Rat

## Abstract

Eggshell membrane intake is considered to have beneficial effects on bone health; however, relevant evidence remains scant. Therefore, we aimed to explore the direct effects of eggshell membrane intake on osteogenic function in normal growing rats. Six-week-old male Wistar rats were divided into control (CO) and eggshell membrane (EM) groups. The experiment was conducted over 8 weeks. Visual observation and micro-computed tomography analysis revealed a significant increase in bone mass in the EM group compared with that in the CO group. Histological analysis showed thick and long trabeculae in the EM group, accompanied by an increase in the number of osteoblasts and suppression of adipocyte accumulation. Furthermore, *Col1a1* expression was significantly higher in the EM group than in the CO group, although no significant differences were found in the number of TRAP-positive osteoclasts or *Ctsk* expression. Immunohistochemical analysis demonstrated a notable increase in the number of Col1-positive osteoblasts but a significant decrease in the number of Dlk1-positive adipocytes in the EM group. Gene expression analysis revealed no difference in the expression of *Runx2* (the master regulator of osteoblast differentiation) between the groups. However, the expression of *Sp7*, which functions downstream of *Runx2*, was significantly upregulated, whereas that of *Pparg*, the master regulator of adipocyte differentiation, was significantly downregulated in the EM group compared with those in the CO group. Overall, the intake of eggshell membranes may enhance osteogenic function and suppress bone marrow adiposity. These findings support the beneficial effects of eggshell membrane intake on bone health.

## Highlights

• We examined the direct effects of eggshell membrane intake on bone tissue.
• Eggshell membrane intake leads to an increase in bone mass.
• Eggshell membrane intake significantly increases the number of osteoblasts.
• Eggshell membrane intake suppresses bone marrow adiposity.
• The eggshell membrane could help support bone health and prevent bone-related diseases.

## Graphical abstract

**Figure:**
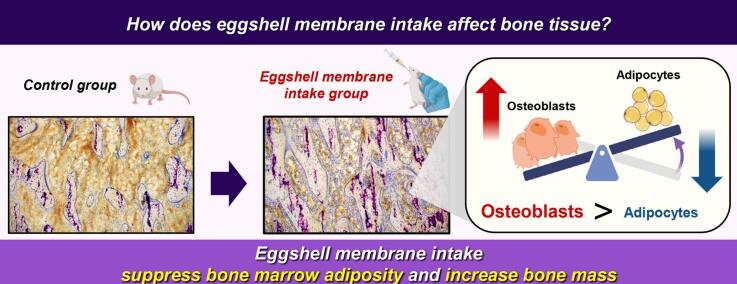

## 1 Introduction

Bone mass is dynamically maintained through a delicate balance between bone formation and resorption, processes that are essential for sustaining bone health (Lerner et al., 2019). When this equilibrium is disrupted, individuals can develop conditions such as osteoporosis, leading to a substantial reduction in bone mass and density. This makes bones more fragile and increases the risk of fractures (Sakat et al., 2018). As bone metabolism slows with age, acquiring and maintaining adequate bone mass become vital for preserving bone strength and preventing fractures (Burr, 2019).

Eggshell membrane is a thin layer lining the inner side of the eggshell, and in recent years, its intake has been noted for potential benefits to bone health (Fladerer and Grollitsch, 2024). The eggshell membrane reportedly exhibits anti-inflammatory properties (Ruff and DeVore, 2014) and antioxidant effects (Sim et al., 2023), both of which are important for maintaining bone health. The eggshell membrane also contains key bone components such as type I collagen (Col1), chondroitin sulfate, dermatan sulfate, osteopontin, and sialoprotein (Hua et al., 2020; Qi et al., 2021; Wu et al., 2004; Yi et al., 2004). Owing to these properties, eggshell membrane has also been widely used as a scaffold for bone regeneration in biomaterial engineering (Torres-Mansilla et al., 2023). In addition to its biomaterial applications, eggshell membrane demonstrates broader benefits for bone health. Liu et al. (2023) demonstrated that the administration of a combination of eggshell calcium and eggshell membrane to calcium-deficient mice resulted in a greater increase in bone mass than administration of eggshell calcium alone, which is known to promote bone formation (Liu et al., 2023; Sakai et al., 2017). This finding suggests that eggshell membrane holds remarkable potential to stimulate bone formation. However, the direct effects of eggshell membrane intake on osteogenic function remain underexplored. Unveiling these unknown effects is of importance for developing new strategies to maintain and enhance bone health. Therefore, in this study, we focused on normal growing rats and aimed to elucidate the direct effects of eggshell membrane intake on osteogenic function through histological and biochemical analyses.

## 2 Materials and methods

This study was conducted in accordance with “ARRIVE Essential 10” of the ARRIVE 2.0 guidelines (Percie du Sert et al., 2020) and was approved by the Animal Experiment Committee of Toyo University (Tokyo, Japan; Approval No. 2023-48). To minimize infection risks, strict hygiene was maintained in the laboratory and by the experimenters throughout the study.

### 2.1 Study design

**Fig. 1:**
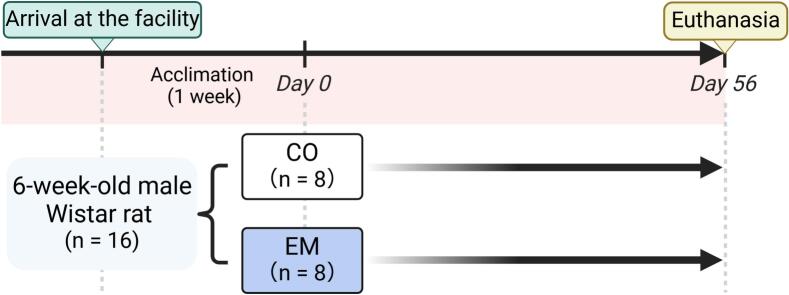
Experimental workflow. All rats were randomly assigned to the EM or CO group after a one-week acclimation period. The EM group was administered 120 mg/kg/day of eggshell membrane orally, five days a week. After the eight-week experiment, all rats were euthanized via carbon dioxide inhalation, and their femurs were collected for analysis.

Six-week-old male Wistar rats (n = 16, weighing 130–150 g; Nippon Bio-Supp. Center, Tokyo, Japan) were used. All rats were delivered under specific pathogen-free conditions and housed in an environment with controlled temperature (24 ± 2 °C) and humidity (50 % ± 5 %), under a 12-:12-h light/dark cycle. The rats were housed in groups of four per cage and provided free access to water and solid feed (Oriental Yeast, Tokyo, Japan). Following a 1-week acclimation period, the rats were randomly assigned to either the eggshell membrane intake group (EM, n = 8) or control group (CO, n = 8) (Fig. 1). Based on a previous study (Sim et al., 2023), rats in the EM group were administered 120 mg/kg/day pulverized eggshell membrane (ALMADO Inc., Tokyo, Japan; Supplementary Table S1) dissolved in water orally via a feeding needle (Primetech, Tokyo, Japan) 5 days a week, in addition to their regular solid feed. The CO group received the same amount of water as the EM group using the same method. No rats were excluded from the study. After 8 weeks, the rats were euthanized via carbon dioxide inhalation, and their femurs were collected for analysis. Among the collected femurs, the right femur was used for micro-computed tomography (µCT) analysis and histological examination, while the left femur was used for gene expression analysis. All experimental results were reviewed by the eight co-authors under blinded conditions to ensure unbiased data interpretation.

### 2.2 μCT analysis

Representative femurs (n = 6) that were not damaged during the sample collection process were used for the final analysis. The femurs were scanned using μCT (SKYSCAN 1276; Bruker, Kontich, Belgium) with the following settings: 70-kV voltage, 57-μA current, and 360° total rotation angle with a rotation step of 0.20°. The acquired scan data were analyzed using the built-in 3D image processing software from SKYSCAN to calculate the bone volume (BV) of the spongiosa.

### 2.3 Tissue processing

The femurs used for μCT analysis were sagittally sectioned along the midline. The medial halves (n = 6) were used to prepare resin-embedded polished sections, while the lateral halves were used for macroscopic observation (n = 3) and to prepare decalcified paraffin-embedded sections (n = 3).

The femurs for macroscopic observation were treated with 3 % sodium hypochlorite solution (195–02206, Wako, Osaka, Japan) to remove soft tissue and then observed.

The femurs used to prepare resin-embedded polished sections were embedded in a mixture resin of Rigolac 2004 (3801; Nisshin EM, Tokyo, Japan) and Rigolac 70F (380; Nisshin EM). The resin blocks were polished to a thickness of approximately 150 μm in the sagittal plane and stained with toluidine blue (209–14,545; Wako).

The femurs for preparing decalcified paraffin-embedded sections were decalcified in 8 % ethylenediaminetetraacetic acid (EDTA, 348–01355; Dojindo, Tokyo, Japan) for 5 weeks and embedded in paraffin (166–18,964; Wako). The paraffin-embedded femurs were sectioned to thin sagittal slices (approximately 4-μm thick) using a microtome (REM-710; Yamato Kohki Industrial, Saitama, Japan).

### 2.4 Quantitative analysis of histological parameters

For the Rigolac specimens (n = 6), the following five parameters were calculated within the defined region of interest (ROI): BV/tissue volume (BV/TV), trabecular thickness (Tb.Th), trabecular number (Tb.N), osteoblast surface/bone surface (Ob.S/BS), and adipocyte number/bone marrow (Ad.N/BM). Ad.N/BM was determined by counting the adipocytes present in the bone marrow, excluding trabecular and vascular spaces.

### 2.5 Tartrate-resistant acid phosphatase (TRAP) staining

Decalcified paraffin-embedded sections were deparaffinized with Lemosol and alcohol and then stained with TRAP staining solution, prepared using naphthol AS-MX phosphate (N5000-1G; Sigma, Tokyo, Japan), fast red violet LB salt (F3381–500MG; Sigma), sodium hydrogen carbonate (191–01305; Wako), and L-(+)-tartaric acid (207–00055; Wako) at 37 °C for 10 min. The sections were counterstained with methyl green (134–13,901; Wako). Using different sections from each group (n = 3), the number of TRAP-positive cells per BS (TRAP-positive cells/BS) within the designated ROI was determined.

### 2.6 Immunohistochemical analysis

Decalcified paraffin-embedded tissue sections were blocked with a blocking reagent (PK-4001; Vector, CA, USA) for 60 min to suppress endogenous enzyme activity. Primary antibodies, anti-Col1 (type I collagen, 1:100, bs-10423R; Bioss, MA, USA) and anti-Delta-like 1 homolog (Dlk1) (1:100, bs-2423R; Bioss), were then applied, and the sections were incubated overnight at 4 °C. The negative control for this analysis lacked the primary antibody; instead, we applied only phosphate-buffered saline (162–19,321; Wako), the primary antibody diluent, in the same amount and duration as in the positive control. Subsequently, a secondary antibody, Goat anti-Rabbit IgG H&L (Alexa Fluor 488) (1:200, ab150077; Abcam, Cambridge, UK), was applied, and the sections were incubated for 60 min. Nuclear staining was performed with 4′,6-diamidino-2-phenylindole (ab104139; Abcam). Different sections (n = 3) were used to determine the number of Col1-positive cells per trabecular surface, and the number of Dlk1-positive cells in the bone marrow was determined in the designated ROI using different sections from each group (n = 3).

### 2.7 Gene expression analysis

The extracted femurs were homogenized under sterile conditions, and total RNA was extracted using TRIzol (15,596,018; Invitrogen, MA, USA). Samples with evident RNA degradation were excluded from the analysis, and the final number of samples analyzed was six (n = 6). The extracted RNA was reverse-transcribed to cDNA using the iScript™ gDNA Clear cDNA Synthesis Kit (1,725,034; Bio-Rad, Tokyo, Japan). The resulting cDNA was mixed with various TaqMan probes, and quantitative real-time polymerase chain reaction (qRT-PCR) analysis was performed. The TaqMan probes (Invitrogen) amplified *Gapdh* (encoding GAPDH; Rn01775763_g1) as the housekeeping gene and *Ctsk* (encoding cathepsin K; Rn00580723_m1), *Col1a1* (encoding Col1; Rn01463848_m1), *Runx2* (encoding Runx2; Rn01512298_m1), *Sp7* (encoding Sp7; Rn02769744_s1), and *Pparg* (encoding Pparg; Rn00440945_m1) as the target genes. qRT-PCR was performed using the CFX96 Touch Real-Time PCR Detection System (47,153; Bio-Rad), and the Ct values for all samples were calculated. Relative gene expression was calculated using the ΔΔCt method (Livak and Schmittgen, 2001).

### 2.8 Statistical analysis

Data were statistically analyzed using SPSS Statistics version 29 (IBM Corp., Armonk, NY, USA). After confirming that the data of both CO and EM groups followed a normal distribution using the Shapiro–Wilk test, an independent *t*-test was performed. Statistical significance was set at p < 0.05.

## 3 Results

**Fig. 2:**
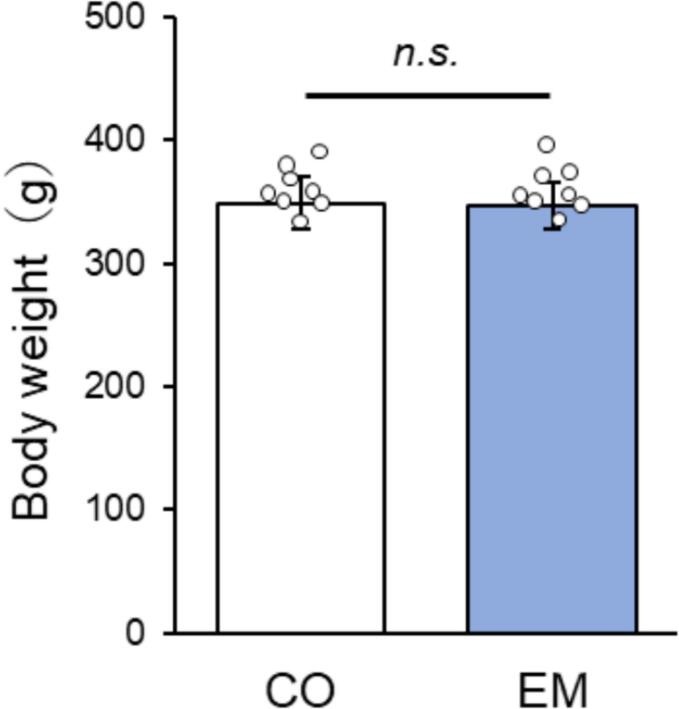
Average body weight of each group (n = 8). Body weight did not differ significantly between the CO and EM groups during the experiment. n.s.: not significant.

No significant differences in body weight were observed between the CO and EM groups throughout the experimental period (Fig. 2), indicating that eggshell membrane intake did not affect weight gain or loss. This finding suggests that there were no health issues.

### 3.1 Eggshell membrane intake increases spongiosa volume

**Fig. 3:**
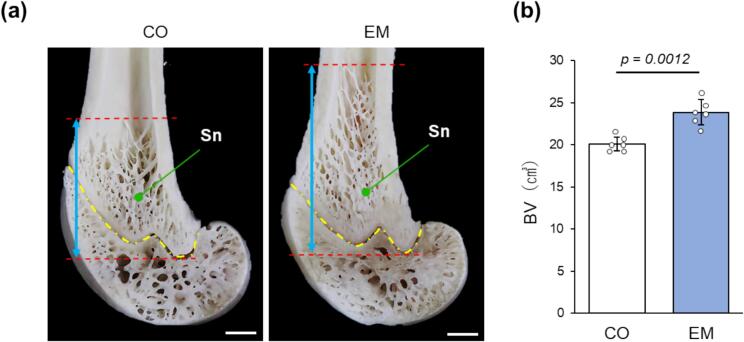
Macroscopic findings and quantification of spongiosa in the distal femoral metaphysis. (a) Macroscopic view of the distal femoral metaphysis. Sn: spongiosa. Blue arrows: Longitudinal width of spongiosa. Yellow line: Growth plate. Red lines: Upper and lower ends of spongiosa. Scale bars = 1 mm. (b) Bone volume (BV) as calculated via μCT analysis (n = 6).

Considerable differences in distal femoral metaphysis were observed between the CO and EM groups during macroscopic analysis (Fig. 3a). The longitudinal width of the spongiosa was greater and the trabeculae appeared more abundant in the EM group than in the CO group. Moreover, the μCT analysis demonstrated that BV in the spongiosa was significantly higher in the EM group than in the CO group (Fig. 3b).

### 3.2 Eggshell membrane intake promotes bone formation and suppresses bone marrow adiposity

**Fig. 4:**
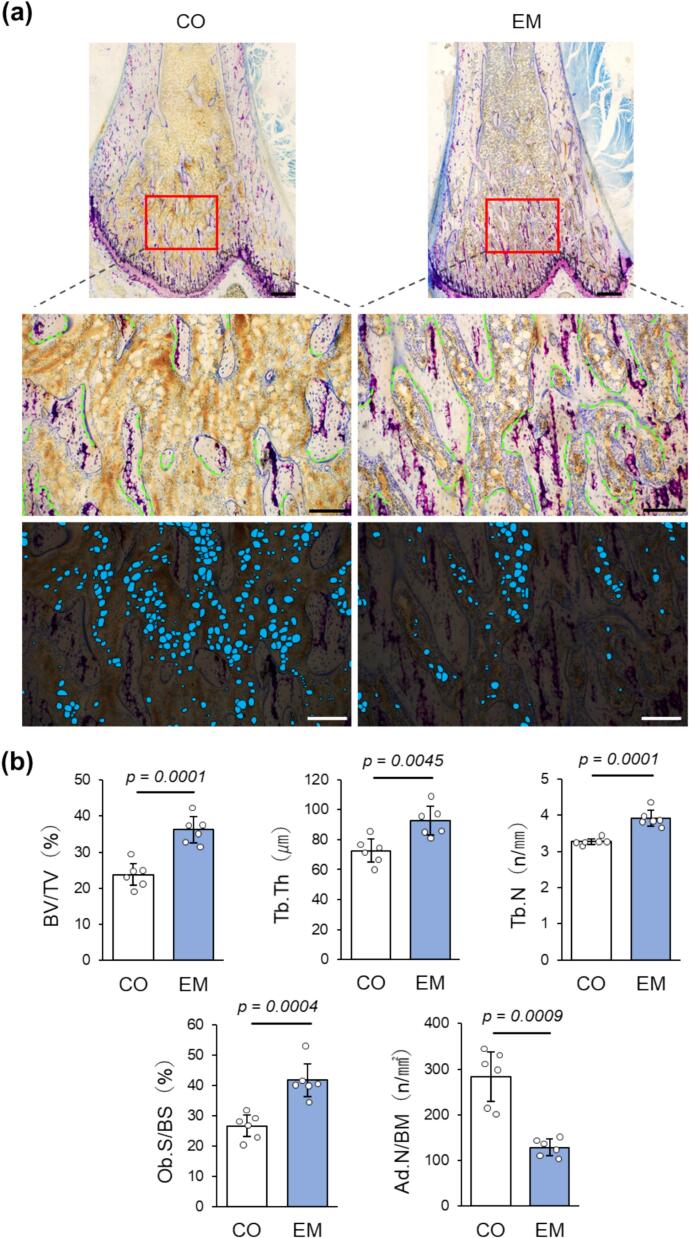
Histological and morphometry. (a) Rigolac specimens of the distal femoral metaphysis (toluidine blue staining). Top: Low magnification (×4), scale bars = 500 μm. Middle: Medium magnification (×10). Green lines: Osteoblast surface, scale bars = 200 μm. Bottom: Trace image of adipocytes (blue) (×10), scale bars = 200 μm. (b) Results of morphometric analysis (n = 6).

The histological analysis of Rigolac specimens stained with toluidine blue supported the macroscopic findings (Fig. 4a). In the EM group, trabeculae were denser, thicker, and extended longer along the longitudinal axis than those in the CO group. Additionally, osteoblast accumulation on the trabecular surface was more pronounced in the EM group than in the CO group. Although adipocytes were present in the bone marrow between the trabeculae in both groups, they were noticeably fewer in the EM group than in the CO group. These histological findings were quantitatively evaluated using morphometry (Fig. 4b). The EM group had significantly higher values for BV/TV, Tb.Th, Tb.N, and Ob.S/BS but significantly lower Ad.N/BM than the CO group.

### 3.3 Eggshell membrane intake does not affect bone resorption

**Fig. 5:**
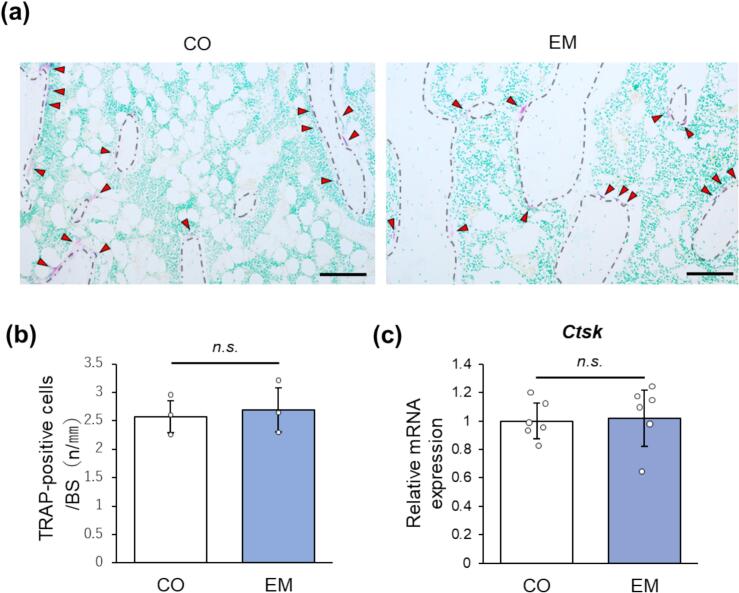
Bone resorption assessment. (a) Histological images of TRAP staining. Methyl green was used as a counterstain. Dashed line: Trabecular surface. Arrowheads: TRAP-positive cells. Scale bars = 100 μm. (b) Quantification of TRAP-positive cells (n = 3). n.s.: not significant. (c) Relative mRNA expression of *Ctsk* (n = 6). n.s.: not significant.

Bone resorption on the trabecular surface was assessed using TRAP staining, a marker for osteoclasts (Wei et al., 2022) (Fig. 5a). TRAP-positive osteoclasts were present on the trabecular surface in both CO and EM groups, and there was no significant difference in their numbers (Fig. 5b). Similarly, no significant difference in *Ctsk* expression was observed between the groups (Fig. 5c).

### 3.4 Eggshell membrane intake promotes osteoblast differentiation and suppresses adipocyte differentiation

**Fig. 6:**
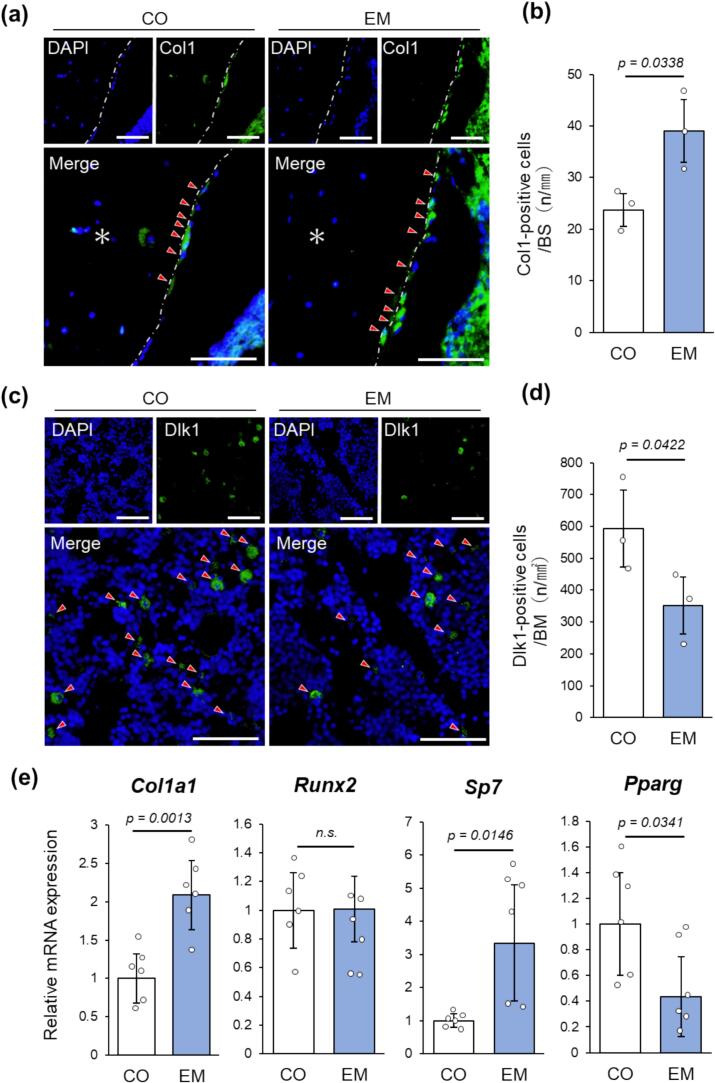
Immunohistochemical and gene expression results. (a) Immunoreactivity of Col1-positive cells. Nuclei were stained with DAPI. Asterisks indicate trabeculae. Scale bars = 50 μm. (b) Quantification of Col1-positive cells (n = 3). (c) Immunoreactivity of Dlk1-positive cells. Nuclei were stained with DAPI. Scale bars = 50 μm. (d) Quantification of Dlk1-positive cells (n = 3). (e) Relative mRNA expression levels of *Col1a1*, *Runx2*, *Sp7*, and *Pparg* (n = 6). n.s.: not significant.

To explore the effects of eggshell membrane intake on the differentiation of osteoblasts and adipocytes, we examined early differentiation markers via immunohistochemistry, using Col1 (Alcantara et al., 2011) as a marker for osteoblasts and Dlk1 (Andersen et al., 2009) as a marker for adipocytes. Although Col1-positive cells were present along the trabecular surface in both CO and EM groups, their numbers were significantly higher in the EM group than in the CO group (Fig. 6a, b). Dlk1-positive cells were found in the bone marrow of both groups, but their numbers were significantly lower in the EM group than in the CO group (Fig. 6c, d). Gene expression analysis revealed significantly higher expression of *Col1a1* and *Sp7* in the EM group than in the CO group, but there was no significant difference in *Runx2* expression between the groups (Fig. 6e). Conversely, *Pparg* expression was significantly lower in the EM group than in the CO group (Fig. 6e).

## 4 Discussion

To the best of our knowledge, this is the first study to examine the direct effects of eggshell membrane intake on osteogenic function. Our study revealed that the longitudinal width of the spongiosa, along with overall bone mass, significantly increased in the EM group. This finding suggests that the intake of eggshell membrane enhances bone formation, supporting previous research findings (Liu et al., 2023). In contrast, under pathological conditions, such as osteopetrosis, excessive bone formation can result in >50 % increase in bone mass (Swarnkar et al., 2015). However, the rate of bone density increase in the EM group in the present study remained below 50 %. This finding indicates that the observed increase in bone mass following eggshell membrane intake is physiological rather than pathological, resulting from enhanced bone formation within the normal range. In addition, previous studies have demonstrated that body weight influences bone mass (Montazerifar et al., 2014; Schafer, 2016). However, the present study revealed no notable differences in body weight between the CO and EM groups, underscoring that the observed increase in bone mass due to eggshell membrane intake occurs independent of body weight.

Bone is a dynamic tissue constantly undergoing remodeling through the coordinated activities of osteoblasts and osteoclasts. This dynamic equilibrium ultimately determines bone mass (Chen et al., 2018). During bone formation, osteoblasts deposit proteins such as Col1, osteocalcin, and alkaline phosphatase onto bone surfaces (Rutkovskiy et al., 2016), and they are later resorbed by osteoclasts using enzymes such as cathepsin K and matrix metalloproteinase 9 (Zhu et al., 2022). In the present study, the EM group exhibited significant accumulation of osteoblasts on trabecular surfaces, whereas the number of osteoclasts, as determined using TRAP staining, remained unchanged. Enhanced osteoblast activity was further evidenced by significantly upregulated *Col1a1* expression in the EM group, whereas *Ctsk* expression did not differ between the groups. These results suggest that the increase in bone mass observed in the EM group is primarily driven by the activation of osteoblast function, with no involvement of increased bone resorption.

The most notable finding of this study was the marked reduction in the number of bone marrow adipocytes in the EM group compared with that in the CO group. Mesenchymal stem cells (MSCs) have the ability to differentiate into either osteoblasts or adipocytes, and their differentiation pathway exhibits a dynamic balance in which the promotion of one pathway suppresses the other (Abdallah and Kassem, 2012). For example, conditions such as aging, osteoporosis, and reduced mechanical loading are known to promote the differentiation of MSCs into adipocytes, leading to increased accumulation of bone marrow fat and a consequent decrease in bone mass (Keune et al., 2017; Woods et al., 2020). Based on these findings, the observed suppression of adipocyte accumulation in the EM group suggests that eggshell membrane intake may influence the differentiation mechanism of MSCs, promoting osteoblast formation while inhibiting differentiation into adipocytes. This hypothesis is strongly supported by the results showing an increase in the number of Col1 (an early osteoblast differentiation marker)-positive cells and a decrease in the number of Dlk1 (an early adipocyte differentiation marker)-positive cells in the EM group. Furthermore, the gene expression analysis revealed significantly suppressed expression of *Pparg* (the master regulator of adipocyte differentiation) in the EM group, further corroborating these findings. In contrast, the expression of *Runx2*, a master regulator of osteoblast differentiation, showed no differences between the groups. However, considering the decreased expression of *Pparg* in the EM group, it can be inferred that the relative expression of *Runx2* was higher in the EM group than in the CO group (Akune et al., 2004; Komori, 2024). Supporting this inference, we observed that the expression of *Sp7*, a transcription factor functioning downstream of *Runx2*, was significantly upregulated in the EM group. Additionally, *Sp7* regulates the expression of numerous bone formation-related genes, including *Col1a1* (Chen et al., 2019; Jiang et al., 2024; Ortuño et al., 2013). Based on these findings, it is suggested that the intake of eggshell membrane primarily promotes the differentiation of MSCs into osteoblasts while suppressing their differentiation into adipocytes, thereby contributing to increased bone mass and suppression of bone marrow adiposity.

A recent review (Fladerer and Grollitsch, 2024) highlighted the similarity in the composition of bone tissues and eggshell membranes as a key factor contributing to the beneficial effects of eggshell membrane intake on bone formation. In contrast, the current study revealed a novel mechanism by which eggshell membrane intake increases bone mass through the regulation of the differentiation mechanisms of osteoblasts and adipocytes. This result suggests that the enhancement of osteogenic function by eggshell membrane intake is due to not only the compositional similarity between eggshell membrane and bone tissue but also its components functioning as bioactive substances. More detailed biochemical analyses in the future will provide valuable insights into the specific underlying mechanisms and pathways.

We have clearly demonstrated through histological evidence that eggshell membrane intake directly affects bone structure and function, but we also acknowledge certain limitations of the study. First, as the primary objective of this study was to elucidate the direct effects of eggshell membrane intake on osteogenic function, we focused on normal growing rats. Hence, we were unable to conduct investigations using commonly employed osteoporosis models, such as aged and ovariectomized models. Further investigations using these models are necessary to clearly demonstrate whether eggshell membrane intake is effective for the prevention or treatment of osteoporosis. Furthermore, the optimal dosage, intake duration, and frequency of eggshell membrane intake were not thoroughly investigated. These factors are critical for verifying the safety and efficacy of eggshell membrane intake and should be evaluated in the future studies in clinical trials.

## 5 Conclusion

We conclude that eggshell membrane intake has the potential to suppress bone marrow adiposity and increase bone mass. These findings serve as fundamental data supporting the beneficial effects of eggshell membrane intake on bone health and provide valuable insights for future research exploring its effectiveness in the prevention and treatment of osteoporosis.

The following is the supplementary data related to this article.

Supplementary Table S1 Composition and characteristics of Eggshell Membrane. Reference data from the certificate of analysis provided by the manufacturer (ALMADO Inc.). Supplementary Table S1

## CRediT authorship contribution statement

**Nao Yashima:** Writing – original draft, Visualization, Validation, Methodology, Conceptualization. **Kaoru Fujikawa:** Writing – review & editing, Conceptualization. **Wataru Minamizono:** Investigation. **Hiroya Matsunaga:** Investigation. **Jiazheng Lyu:** Investigation. **Hirai Suito:** Writing – review & editing. **Takumi Okunuki:** Writing – review & editing. **Shingo Nakai:** Writing – review & editing. **Masafumi Ohsako:** Supervision, Project administration, Funding acquisition.

## Funding

This work was supported by ALMADO Inc.

## Declaration of competing interest

The authors declare that they have no competing interests.

## Data Availability

The data that support the findings of this study are available from the corresponding author on reasonable request.
