# Supplementary material for: Intake of eggshell membrane enhances bone mass and suppresses bone marrow adiposity in normal growing rats
Source: Bone Rep. 2025 Apr 1;25:101840. doi: 10.1016/j.bonr.2025.101840 (PMC11999470; doi:10.1016/j.bonr.2025.101840)
Supplement: Supplementary Table S1 — Composition and characteristics of Eggshell Membrane. Reference data from the certificate of analysis provided by the manufacturer (ALMADO Inc.). [file mmc1.docx]

**Composition and Characteristics of Eggshell Membrane Powder Ingredients**

| Parameter | Content | Test Methods | Remarks |
| --- | --- | --- | --- |
| Energy | 374kcal/100g |  | 1 |
| Moisture | 3.1g/100g | Normal pressure heating drying method |  |
| Total protein | 90.3g/100g | Kjeldahl method |  |
| Total fat | 1.4g/100g | Acid decomposition method | 2 |
| Carbonhydrates | 0g/100g | - |  |
| Total ash | 5.2g/100g | Direct ashing method |  |
| Sodium | 21.9mg/100g | Atomic absorption spectrometry (AAS) |  |
| Salt equivalent amount | 0.06g/100g | - |  |

Remarks

1. * by formula set by Japanese Food Labeling Act (Atwater coefficient factor)
 Energy conversion factor: protein 4kcal/g, fat 9kcal/g, carbohydrates 4kcal/g

2. * by formula set by Japanese Food Labeling Act.
 100 - (moisture + total protein + fat + ash)
